# Supplementary figures and images for: Advancing the communication of genetic risk for cardiometabolic diseases: a critical interpretive synthesis
Source: BMC Med. 2023 Nov 13;21:432. doi: 10.1186/s12916-023-03150-9 (PMC10641935; doi:10.1186/s12916-023-03150-9)

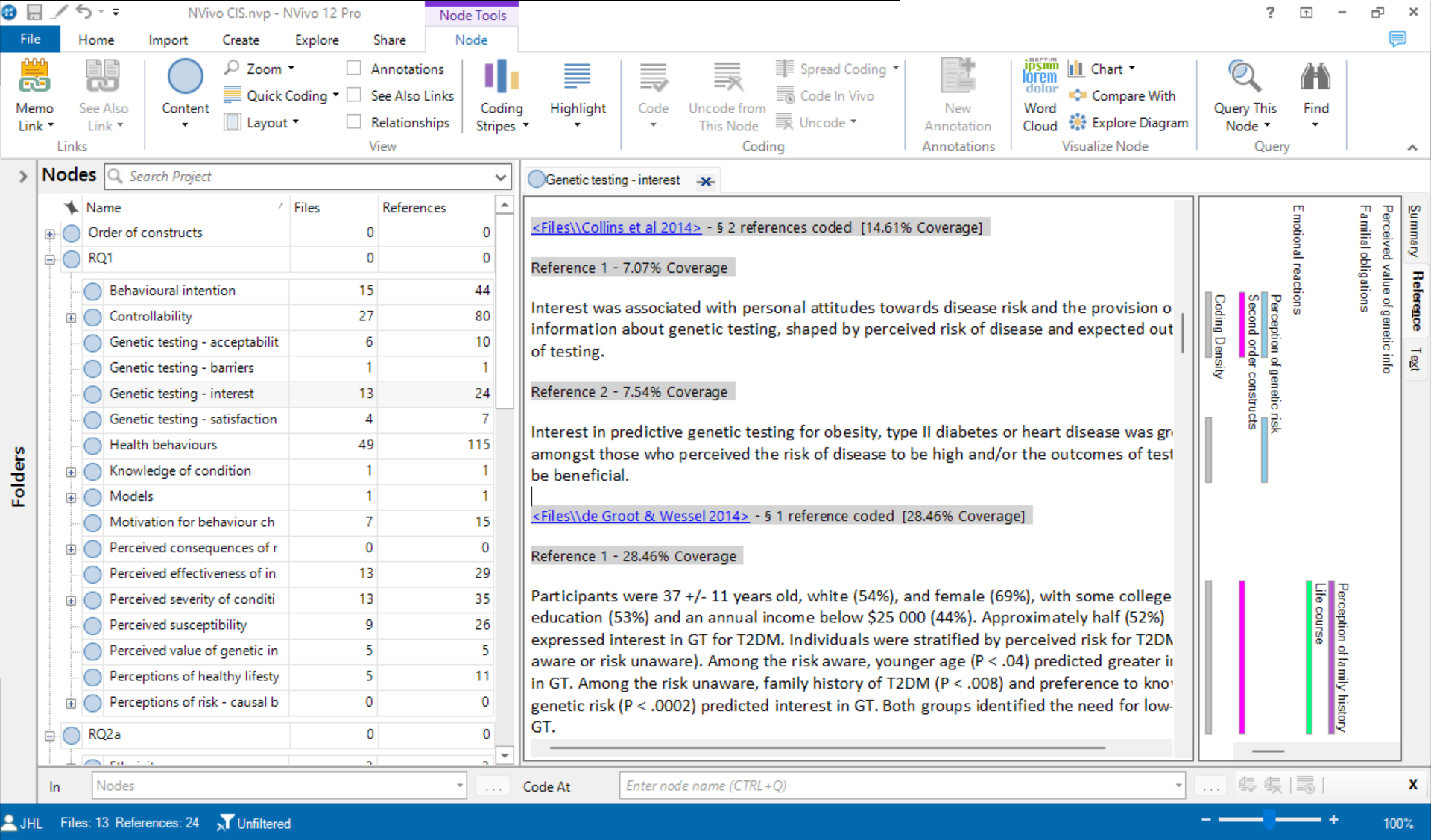

Supplement: Supplementary file 3 — Additional file 3. Example of data extraction process on NVivo. [file 12916_2023_3150_MOESM3_ESM.docx]
